# PLA/Starch Composites: New Applications as Control Release Materials

**DOI:** 10.3390/foods15030454

**Published:** 2026-01-27

**Authors:** Zhibo Zhao, Yanan Li, Yunlong Xu, Jun Fu, Qingfei Duan, Zhenggui Wu, Muzaffar Makhkamov, Amjad Ali, Hongsheng Liu, Long Yu

**Affiliations:** 1 Institute of Chemistry, Henan Academy of Science, Zhengzhou 450002, Chinaxylong@hnas.ac.cn (Y.X.); fujun@hnas.ac.cn (J.F.);; 2 School of Chemistry, Henan University, Zhengzhou 450001, China; 3 School of Materials Science and Engineering, Zhengzhou University, Zhengzhou 450001, China; 4 Department of Polymer Chemistry, National University of Uzbekistan, Tashkent 100174, Uzbekistan; muz_m77@mail.ru; 5 Department of Agriculture and Food Technology, Karakorum International University, Gilgit 15100, Pakistan; dr.amjadali@kiu.edu.pk; 6 School of Food Science and Engineering, South China University of Technology, Guangzhou 510640, China; liuhongsheng@scut.edu.cn

**Keywords:** PLA, starch, composites, control release

## Abstract

Poly(lactic acid) (PLA)/starch composites have attracted considerable attention as promising eco-friendly materials due to their renewable origins and complementary properties. The system synergized benefits including cost reduction and enhancing biodegradation through filled with starch, and reducing moisture sensitivity by adding PLA. In recent years, PLA/starch composites have also emerged as functional materials for controlled-release applications, benefiting from their inherent phase-separated structures and distinct water solubility and degradation behaviors of the two components. By tailoring starch content and dispersion, starch-rich domains can serve as water-responsive pathways within the PLA matrix, enabling tunable release of functional substances from films or coatings. This concept has been successfully demonstrated in applications such as antimicrobial food packaging and slow-release fertilizer coatings. This review first outlines the fundamental aspects of PLA/starch composites, including microstructure, interfacial compatibility, and biodegradability. It then focuses on their design and performance as controlled-release systems, covering fabrication strategies, structure–property relationships, and evaluation methods. Finally, the advantages and limitations of current PLA/starch-based controlled-release materials are critically discussed, and future research directions are proposed to guide the development of sustainable, multifunctional materials for food packaging and agricultural applications.

## 1. Introduction

Bio-based polymers derived from renewable resources have attracted increasing interest as environmentally benign alternatives to conventional petroleum-based plastics. Despite their sustainability advantages, widespread application remains constrained by limitations such as high production cost, inherent brittleness, and limited processability [1,2]. However, most biopolymer blends, including poly(lactic acid) (PLA) and starch, are thermodynamically immiscible, necessitating careful control of blend morphology and interfacial interactions to achieve acceptable performance. Among various strategies, polymer blending and compositing provide a practical and economically viable approach to combining the desirable properties of different biopolymers. Notably, PLA and starch, two biodegradable polymers sourced from abundant feedstocks, have been extensively studied due to their complementary advantages. Starch incorporation can significantly reduce material cost and enhance biodegradability, while PLA contributes to improved moisture resistance and mechanical integrity [3,4,5,6,7]. However, the intrinsic incompatibility between hydrophobic PLA and hydrophilic starch often leads to weak interfacial adhesion and pronounced phase separation, which severely compromise the mechanical and barrier properties of the resulting composites [8,9,10]. Understanding and regulating the structural origins of this incompatibility therefore represent a central challenge in the development of PLA/starch composites.

PLA is one of the most extensively studied bio-based aliphatic polyesters, typically synthesized from lactic acid derived via the fermentation of renewable resources such as starch [11,12,13]. Owing to its biocompatibility, renewability, ease of processing, and potential for thermal and mechanical modification, PLA has been widely applied in packaging, agriculture, and biomedical materials. Its biodegradability further supports its appeal as a sustainable alternative to petrochemical-derived plastics. However, their degradation under natural conditions is often limited, as it requires elevated temperature and humidity to achieve efficient decomposition [14,15,16]. In many cases, complete mineralization may take several months to years, especially in soil environments lacking industrial composting parameters. This slow degradation rate, combined with the relatively high production cost of PLA, restricts its widespread application in cost-sensitive or environmentally demanding scenarios [17,18,19,20,21]. Blending PLA with low-cost, hydrophilic fillers such as starch has emerged as an effective strategy to not only reduce production costs but also enhance environmental degradability. Starch promotes microbial colonization and facilitates water uptake, thereby accelerating the hydrolysis and microbial breakdown of the composite matrix [4,17,21,22,23,24,25,26].

Starch is one of the most attractive natural polymers owing to its inherent biodegradability, low cost, wide availability, and annual renewability. Structurally, starch is a heterogeneous polysaccharide composed primarily of two distinct macromolecular components: amylose, a mostly linear polymer of α-1,4-linked glucose units, and amylopectin, a highly branched polymer containing α-1,4-linked chains with frequent α-1,6 branch points [27]. Beyond its chemical composition, starch exhibits a complex semi-crystalline and hierarchical organization, arising from the packing of amylose and amylopectin double helices, which plays a decisive role in its physicochemical behavior. Depending on botanical origin and hydration state, native starch granules exhibit distinct X-ray diffraction patterns, conventionally classified as A-, B-, or C-type starches. A-type starches, typically found in cereal starches, possess densely packed orthorhombic unit cells, whereas B-type starches, commonly associated with tuber and high-amylose starches, exhibit more open monoclinic unit cells with higher water content and enhanced swelling ability. C-type starches represent mixed crystalline forms containing features of both A- and B-type structures. These differences in molecular organization strongly influence water uptake, swelling behavior, gelatinization, and mechanical response, which are critical for starch processing and performance in polymer composite systems [28].

**Figure 1:**
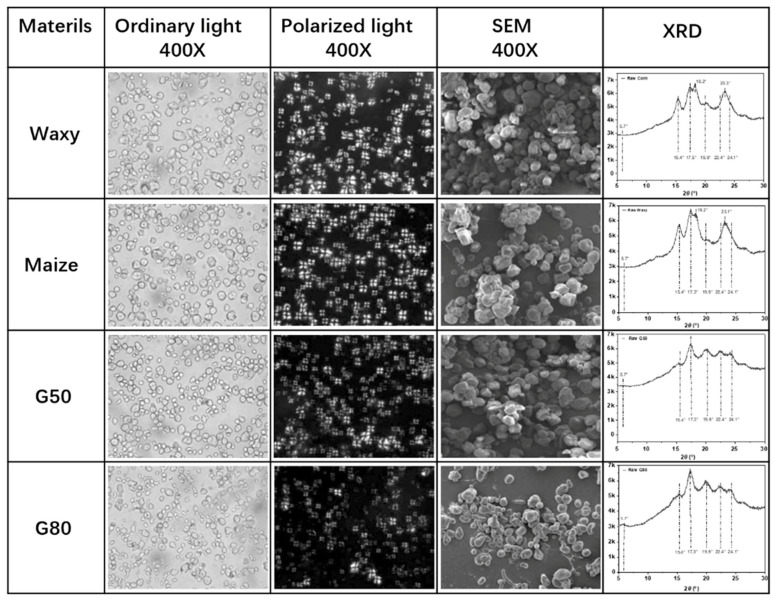
Morphologies of cornstarch with different amylose/amylopectin ratios and their crystal structures detected by XRD [35].

As a result, the physicochemical properties of native starches, including granule size, crystallinity, thermal stability, and moisture sensitivity are strongly governed by their amylose-to-amylopectin ratio and crystalline polymorphism. The hydrophilic nature of starch arises from its high density of hydroxyl groups, including secondary hydroxyls at the C-2 and C-3 positions and a primary hydroxyl group at the C-6 position of each glucose residue. These functional groups confer strong water affinity, but also render starch-based materials highly moisture-sensitive and mechanically weak, limiting their standalone use in structural applications [29,30,31,32,33,34,35]. As shown in Figure 1, cornstarches with different amylose/amylopectin ratios exhibit distinct surface morphologies and X-ray diffraction (XRD) patterns, reflecting differences in molecular packing and internal order. Waxy starch and normal maize starch exhibit diffraction features commonly associated with A-type XRD classification patterns, characterized by pronounced reflections near 2*θ* ≈ 15.4°, 17.5°, 18.2°, and 23.3°, and relatively higher apparent crystallinity values of approximately 34.2% and 30.3%, respectively [36,37]. In contrast, starches with higher amylose content, such as G50 and G80, display diffraction signatures typically attributed to B-type classification patterns, including reflections near 2*θ* ≈ 17.5°, 19.9°, 22.4°, and 24.1°, accompanied by a weakened feature around 2*θ* ≈ 15.4°, and lower apparent crystallinity values of approximately 19.1% and 17.8%, respectively [38,39]. It should be noted that the positions, intensities, and shapes of diffraction peaks are influenced by multiple factors, including hydration level, botanical origin, crystallite size, and preferred orientation.

Despite these intrinsic limitations, starch remains a highly attractive filler or matrix component in biodegradable composite systems, particularly when combined with thermoplastic polyesters such as PLA. Its low cost, wide availability, and compatibility with conventional melt-processing techniques make starch an effective route to reduce the overall cost of bio-based materials [4,7,40,41,42,43,44,45,46,47]. Nevertheless, achieving a balance between mechanical performance, moisture resistance, and processability remains challenging due to starch’s brittleness and water sensitivity [1,7,48,49,50]. Recent studies have demonstrated that these challenges can be mitigated through compositional optimization, interfacial modification, and processing innovations, leading to improved performance of PLA/starch composites [3,51,52,53,54]. For example, Khalid et al. [15] conducted a systematic investigation into the effects of starch granule microstructure and morphology on the mechanical and interfacial properties of PLA/starch composites. Their findings revealed that (a) well-dispersed, small-sized starch granules or those with high amylose content (e.g., G-50 and G-80) significantly enhanced the reinforcement effect while minimizing deformation loss; (b) surface modification of starch effectively suppressed granule aggregation and improved dispersion within the PLA matrix; and (c) the botanical source of starch had negligible impact on the thermal stability of the composites. In a separate study, Duan et al. [55] reported one-step extrusion to minimize thermal decomposition for processing PLA/starch composites, in which a side feeder used for adding starch and a sheet die at end of extruder were designed and applied for fabrication. These advances, together with a growing number of patents and review articles, underscore the increasing interest in and technical potential of PLA/starch composites as sustainable and multifunctional material platforms.

In view of the growing interest in sustainable food-related materials, this review focuses on PLA/starch composites from a structure–function perspective, with particular emphasis on their relevance to food packaging and controlled-release systems. Special attention is given to the emerging use of PLA/starch composites as controlled-release platforms, where phase-separated morphologies and water-responsive starch domains play a critical role in regulating the release of active substances in antimicrobial packaging and related applications. The review is organized as follows: fundamental aspects of PLA/starch composites, including material characteristics and compatibility issues, are first introduced; fabrication strategies and microstructural features are then discussed in relation to functional performance; finally, recent advances in controlled-release applications, along with current challenges and future research directions, are critically evaluated.

## 2. Interphase and Compatibility of PLA/Starch Composites

Polymer blending is a widely adopted and cost-effective strategy to tailor material properties by combining the advantages of different polymers. However, most polymer pairs, including PLA and starch, are thermodynamically immiscible owing to their distinct polarity, molecular structure, and intermolecular interactions. As a result, phase separation and weak interfacial adhesion are commonly observed in PLA/starch blends, which negatively affect mechanical integrity as well as barrier performance. In these systems, starch is typically introduced as a low-cost and biodegradable filler; nevertheless, its incorporation often leads to deterioration of structural properties due to insufficient interfacial compatibility. These interfacial deficiencies directly translate into compromised mechanical strength, barrier performance, and moisture sensitivity, which in turn dictate the functional limitations and opportunities discussed in later sections. Therefore, achieving an appropriate balance between economic feasibility and functional performance remains a central challenge in the development of PLA/starch composites.

Yu et al. [1] reviewed various polymer blends and composites derived from renewable resources including PLA/starch systems, and introduced various methodologies to improve their interfaces. In general, biodegradable aliphatic polyesters such as PLA are intrinsically incompatible with starch because of the hydrophobic nature of PLA and the hydrophilic character of starch. From a thermodynamic perspective, fully miscible polymer blends are rare, as the Gibbs free energy of mixing is typically positive. This arises from the negligible entropy gain associated with polymer–polymer mixing and a positive enthalpy contribution resulting from unfavorable intermolecular interactions. Consequently, interfacial engineering is essential for improving the dispersion and adhesion between PLA and starch phases. To address these challenges, a variety of compatibilization and interfacial modification strategies have been developed [9,56,57].These approaches mainly include: (i) grafting hydrophilic moieties (e.g., maleic anhydride) onto PLA chains to enhance hydrogen bonding and wettability [58,59,60]; (ii) introducing hydrophobic functionalities onto starch backbones to improve its affinity with PLA [10,61,62], and (iii) employing reactive coupling agents, such as methylene diphenyl diisocyanate (MDI), to form covalent linkages between PLA and starch [57,63]. These strategies aim to reduce interfacial tension, suppress phase separation, and promote stress transfer across the interface.

It is known that the hydrophilic nature of biopolymers, such as polysaccharides, leads to their low mechanical and water resistance properties. Thus, like many other biopolymers, PLA may require some additional processing or modification to develop useful antimicrobial materials. This is primarily due to the intrinsic properties of unmodified PLA, which include high brittleness, a poor water-vapor barrier, low crystallinity, a slow biodegradation rate, hydrophobicity, and a lack of reactive side-chain groups. Several types of modification have been developed to address these inadequacies and include chemical modification (for example, grafting, polymerization), the addition of plasticizers (to reduce brittleness), blending with other biopolymers or fibers, and the addition of compatibilizers to enhance its miscibility with otherwise incompatible polymers [1,64,65,66,67,68,69].

**Figure 2:**
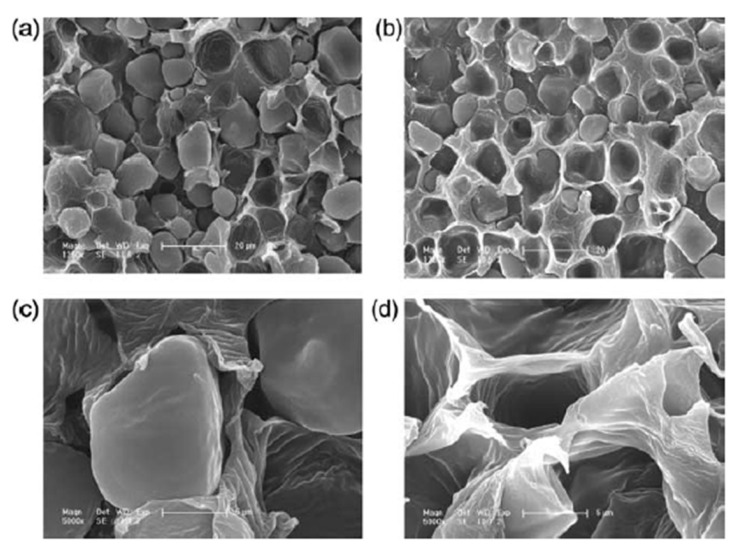
SEM micrographs of the PLLA/starch blends: (**a**) simple PLLA/starch (1:1) blend; (**b**) PLLA/starch/2% St-g-PLLA; (**c**) the magnified image of (**a**); (**d**) the magnified image of sample (**b**) [70].

Despite these efforts, conventional blending or additive-based approaches often lead to only limited improvements in interfacial adhesion and mechanical properties. In contrast, reactive compatibilization strategies, where functional copolymers are generated in situ during melt processing, have shown superior effectiveness in enhancing phase dispersion and interfacial cohesion. Figure 2 illustrates the morphological evolution of PLA/starch composites before and after the introduction of a compatibilizer, specifically starch grafted with poly(L-lactic acid) (St-g-PLLA), as revealed by scanning electron microscopy (SEM) [70]. In the unmodified blend, distinct phase separation is evident, with clear boundaries between starch granules and the surrounding PLA matrix (Figure 2a,c), indicating poor interfacial adhesion. In contrast, the addition of St-g-PLLA results in a smoother and more integrated interface with significantly reduced interfacial voids (Figure 2b,d). This morphological improvement confirms that the compatibilizer effectively promotes molecular-level interactions at the interface, leading to enhanced miscibility and improved interfacial bonding. Smaller or more regularly shaped granules generally promote improved dispersion and stress transfer, whereas larger or irregular starch granules may induce stress concentration, interfacial voids, and preferential water pathways, thereby influencing mechanical performance, barrier properties, and controlled-release behavior. Importantly, while interfacial incompatibility is traditionally regarded as a drawback, the resulting phase-separated morphology can also be deliberately exploited to create water-responsive transport pathways, forming the structural basis for the controlled-release functions discussed in subsequent sections.

## 3. PLA/Starch Composites Used for Antimicrobial Packaging

### 3.1. Overview of Antimicrobial Strategies in PLA-Based Packaging

The development of antimicrobial packaging materials is a rapidly evolving field driven by the growing need to ensure food safety, extend shelf life, and reduce reliance on synthetic preservatives. PLA, owing to its biodegradability, transparency, and processability, has attracted considerable attention as a base material for active packaging. However, native PLA lacks intrinsic antimicrobial activity, necessitating the incorporation of functional agents to inhibit microbial growth [71,72,73,74,75,76,77,78]. Traditionally, antimicrobial packaging strategies have relied on the incorporation of inorganic agents such as zinc, copper, silver, and their oxides. While effective, these substances present significant concerns regarding toxicity, regulatory compliance, and potential migration into food products. As a result, there has been a notable shift toward natural and generally recognized as safe (GRAS) alternatives that offer both efficacy and biocompatibility [79,80,81,82].

Naturally derived antimicrobial agents, including essential oils, plant extracts, polyphenols, and bacteriocins (e.g., nisin), have demonstrated promising potential for safe use in PLA-based food packaging [7,82,83,84,85,86,87,88,89,90,91,92]. These agents act via various mechanisms, such as membrane disruption, oxidative stress induction, and enzyme inhibition, and are generally considered more acceptable by consumers and regulators due to their origin and safety profiles. For example, Del Nobile et al. [93] incorporated lemon extract, thymol, and lysozyme into PLA matrices via melt extrusion. The resulting films exhibited pronounced antimicrobial activity against foodborne pathogens, demonstrating the feasibility of embedding natural bioactives directly into polymeric packaging. Similarly, Jin and Zhang [74] developed PLA/nisin composite films that effectively inhibited the growth of Listeria monocytogenes in both brain heart infusion (BHI) broth and liquid egg white, achieving microbial reductions of up to 50% compared to the control. These studies demonstrate the feasibility of embedding natural bioactives into PLA matrices; however, they also underscore the importance of controlling the stability and release behavior of such agents during storage and use.

More recently, Purmana et al. [94] have systematically summarized progress in bio-based antimicrobial packaging, highlighting not only the diversity of natural antimicrobial compounds but also the technical challenges associated with tailoring packaging films for specific food systems, ensuring industrial scalability, and minimizing environmental impact. These analyses emphasize that antimicrobial efficacy is governed not only by the intrinsic activity of the bioactive compounds, but also by their dispersion, compatibility, and release kinetics within polymer matrices. Among the natural antimicrobials studied, pomegranate peel (PGP) extract has emerged as a particularly promising candidate [95]. As a by-product of the juice and food-processing industries, PGP represents an abundant and low-cost source of bioactive phenolic compounds, including ellagic acid, ellagitannins, catechin, rutin, punicalagin, lignin, and epicatechin [96,97,98,99,100,101]. These compounds exhibit broad-spectrum antimicrobial activity and antioxidant properties, making PGP an attractive functional additive in food packaging applications [102]. However, two major challenges currently limit its widespread use in polymer-based packaging systems: (1) the relatively high cost associated with extraction and purification of active components, and (2) the difficulty of achieving effective dispersion, stabilization, and controlled release of liquid-phase bioactives within solid polymer matrices such as PLA. These limitations necessitate the development of advanced strategies for enhancing the compatibility and release kinetics of PGP-based antimicrobial systems.

Overall, the integration of antimicrobial agents into PLA-based systems represents a promising strategy for active packaging. Continued innovation in the selection of bioactive agents, compatibility enhancement, and release modulation will be critical to advancing this field toward commercially viable, sustainable food packaging solutions. While a wide range of natural antimicrobial agents have been successfully incorporated into PLA-based packaging, achieving stable dispersion and sustained antimicrobial activity remains challenging. In this context, the introduction of starch as a secondary biopolymer has emerged as an effective strategy to modulate matrix hydrophilicity, microstructure, and release behavior, laying the foundation for the functional design of PLA/starch antimicrobial packaging composites.

### 3.2. Functional Design of PLA/Starch Antimicrobial Composites

The incorporation of starch into PLA-based matrices has been extensively investigated as an effective route to develop cost-efficient, biodegradable antibacterial food packaging materials. In addition to reducing formulation cost, starch can enhance biodegradability and promote the formation of heterogeneous and, in some cases, porous microstructures, which in turn influence mass transport and the release kinetics of incorporated antibacterial agents. 

Various biobased fillers and plasticizers have been incorporated into PLA/starch systems to improve the antimicrobial efficacy and modulate release behavior. Among these, lignocellulosic materials, wood flour, and polysaccharide derivatives are frequently utilized due to their affinity with hydrophilic antimicrobial agents. For example, Prapruddivongs and Sombatsompop [103] investigated PLA/wood flour composites loaded with triclosan and found that increasing wood flour content promoted the migration of triclosan toward the film surface, significantly improving the inhibition of Escherichia coli. This effect was attributed to the increased porosity and surface roughness imparted by the lignocellulosic filler, which facilitated moisture uptake and diffusion of the active compound. Liu et al. [77] further demonstrated that a neat PLA film containing low concentrations of Nisin did not exhibit effective antimicrobial activity. However, when co-extruded with pectin–Nisin complexes, the resultant composite film displayed significantly enhanced antibacterial performance against Listeria monocytogenes. The protective matrix formed by pectin was found to preserve the bioactivity of the loaded agent and facilitate its release, underscoring the role of hydrophilic biopolymers in controlled-release systems.

Beyond conventional hydrophilic fillers, nanostructured additives have been employed to improve dispersion and release performance. Rhim et al. [104] demonstrated that PLA/nanoclay composites prepared using Cloisite 30B exhibited enhanced antimicrobial properties, likely due to increased interfacial area and water permeability. Meanwhile, Kayaci et al. [105] developed electrospun PLA nanofiber mats incorporating triclosan–cyclodextrin inclusion complexes. The resulting materials displayed larger inhibition zones against both E. coli and Staphylococcus aureus, attributed to the synergistic effects of nanofiber morphology and sustained release from the inclusion complexes. Moreover, Ramos et al. [106] incorporated thymol and silver nanoparticles into PLA films to achieve dual antioxidant and antimicrobial functions. The modified films not only improved bacterial inhibition but also showed enhanced disintegration under composting conditions, demonstrating the potential of combining natural bioactives with nano-reinforcements to achieve both functionality and degradability. Table 1 summarizes representative antibacterial agents incorporated into PLA-based composite systems.

Recent studies have further demonstrated that PLA/starch-based composite architectures (e.g., multilayer or bilayer designs) can be tailored for specific food packaging scenarios by regulating moisture and gas transport. Zhou et al. [109] developed pea starch/PLA bilayer films for cherry tomato packaging; the layered structure synergistically regulated moisture and gas exchange, effectively delaying fruit softening, discoloration, and decay, and extending shelf life by 3–5 days relative to conventional packaging. Gui et al. [110] incorporated modified porous starch into PLA/poly(butylene adipate-co-terephthalate) (PBAT) blends, enabling sustained release as well as antimicrobial and antioxidant functions. Compared with unmodified PLA/PBAT films, the active films reduced microbial counts on packaged meat by 62% and inhibited lipid oxidation by 48%, which is particularly important for suppressing spoilage in high-fat foods. Natural reinforcements have also been explored to enhance performance and functionality. Freitas et al. [111] used rice straw fractions to reinforce PLA/starch bilayers for meat preservation; the lignocellulosic components increased tensile modulus by 29% and synergistically suppressed microbial growth and lipid oxidation, reducing the rancidity rate of refrigerated pork by 45% compared with neat PLA films. For targeted antibacterial and antioxidant functions, Muller et al. [112] reported PLA/starch bilayer films loaded with cinnamaldehyde, which combined controlled release with strong antimicrobial efficacy, inhibiting typical spoilage microorganisms such as Escherichia coli and Aspergillus niger on fresh vegetables and reducing colony counts by more than 60% compared with films without cinnamaldehyde. Table 2 summarizes food packaging applications PLA/starch-based composite films.

**Figure 3:**
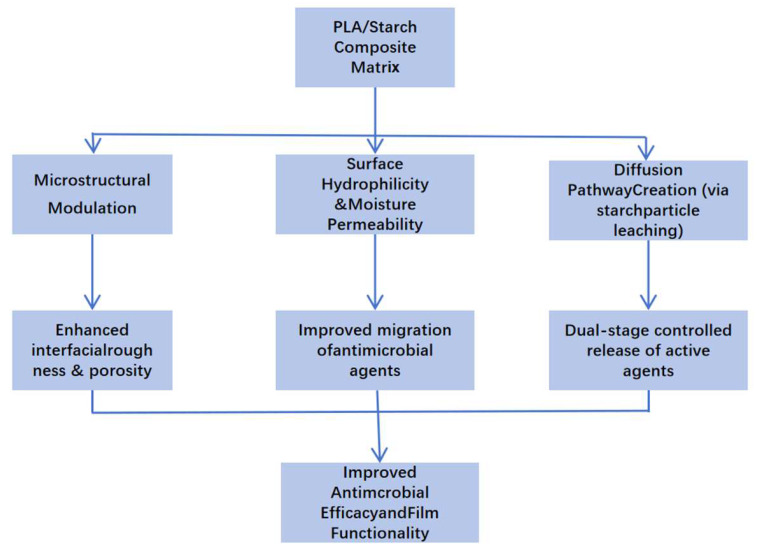
Schematic illustration of the role of starch in modulating the structural, physicochemical, and functional properties of PLA-based antimicrobial composites.

Overall, the functional performance of PLA/starch antibacterial food packaging composites is governed by the ability of starch domains to act as water-triggered release pathways within the PLA matrix. Upon moisture exposure, hydrophilic starch-rich regions promote water uptake, microchannel formation, and controlled diffusion of antibacterial agents, enabling sustained surface activity. Figure 3 schematically illustrates the multifunctional roles of starch in regulating the structural, physicochemical, and functional properties of PLA-based antibacterial composites. This structure-enabled release behavior provides the foundation for the controlled-release mechanisms discussed in the following section.

Collectively, these studies emphasize that the functional design of PLA/starch antimicrobial composites requires careful selection and engineering of filler morphology, interfacial interactions, and hydrophilic–hydrophobic balance. Starch plays a pivotal role in modulating the structural and transport properties of the matrix, which are critical for achieving desired antimicrobial release profiles and overall material performance. Table 1 shows the summary of antimicrobial agents incorporated in PLA-based composite systems. Figure 3 shows the schematic illustration of the role of starch in modulating the structural, physicochemical, and functional properties of PLA-based antimicrobial composites. Starch serves multiple functional roles in PLA-based antimicrobial composite systems. First, starch granules disrupt the homogeneity of the PLA matrix, introducing microstructural heterogeneities such as pores, interfacial roughness, and microchannels. Second, the hydrophilic nature of starch increases the matrix’s surface wettability and water uptake, which is critical for activating and transporting embedded antimicrobial compounds. Third, upon contact with moisture, starch can partially dissolve, forming pathways that facilitate controlled diffusion of active agents. These combined effects result in enhanced migration, sustained release, and higher surface activity of antimicrobial agents, thereby improving the overall functional performance of the composite packaging material.

### 3.3. Controlled Release Mechanisms in PLA/Starch-Based Systems

From a structural perspective, the controlled release behavior arises from the synergistic contrast between the relatively crystalline PLA matrix, which acts as a diffusion barrier, and the starch-rich domains, which become hydrated or partially dissolved upon moisture exposure to form preferential release channels for antimicrobial agents. Building on the role of starch-rich domains as water-triggered release pathways in PLA/starch antibacterial food packaging, the controlled-release behavior of these systems is primarily governed by: (1) Moisture Penetration: Upon exposure to humid environments, hydrophilic starch domains preferentially absorb water, which initiates the swelling process and the formation of interconnected microchannels within the PLA matrix. (2) Swelling and Partial Dissolution of Starch: As starch swells or partially dissolves, it alters the matrix structure, creating pathways that facilitate the diffusion of antimicrobial agents. (3) Initial Burst Release: Water-soluble or surface-localized antibacterial agents are released rapidly upon water uptake, providing an immediate antimicrobial effect. (4) Sustained Diffusion-Controlled Release: After the burst release, the remaining antimicrobial agents diffuse slowly through the PLA matrix, regulated by water absorption and transport through the hydrophobic PLA matrix. (5) Influence of Interfacial Boundaries: Phase-separated morphologies and interfacial boundaries between PLA and starch further modulate the release rate, directionality, and uniformity of agent transport, contributing to the sustained antimicrobial activity. Together, these mechanisms underpin the effectiveness of PLA/starch composites as controlled-release platforms for food packaging applications [117].

**Figure 4:**
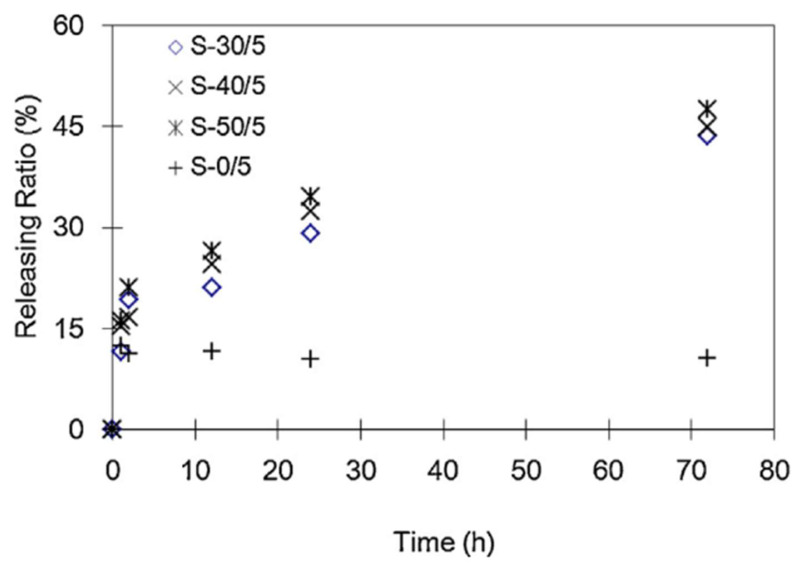
The chitosan releasing ratio in starch/PLA antimicrobial materials as a function of time [113].

Hydrophilic or porous fillers, such as starch, have been widely employed to tailor the release profiles of antimicrobial compounds in PLA-based matrices. For example, Prapruddivongs and Sombatsompop [103] demonstrated that wood flour in PLA composites acted not only as a reinforcing filler but also as a release promoter for triclosan. Increasing wood flour content significantly enhanced the migration of triclosan toward the film surface, thereby improving antibacterial efficacy. This behavior was attributed to increased porosity and moisture uptake, which facilitated diffusion of the active agent. Similarly, Liu et al. [78] developed a slow-release antimicrobial system based on PLA/starch/chitosan ternary blends, in which chitosan served as the active compound while PLA and starch formed the release-controlling matrix. The study revealed a two-phase release mechanism: an initial burst attributed to surface-bound chitosan, followed by a prolonged diffusion-controlled release as water penetrated the hydrophilic starch domains. Increasing starch content enhanced the hydrophilicity of the blend, promoting water uptake and facilitating sustained release. This dual-stage release profile was particularly effective for food products with high moisture activity, such as fresh meat. Comparable results were reported by Bie et al. [107] who investigated PLA/starch/chitosan composites with varying starch contents. As shown in Figure 4, PLA/chitosan systems without starch (S-0/5) exhibited a rapid initial release of approximately 15% within the first hour, followed by negligible further diffusion, due to limited water permeability of the hydrophobic PLA matrix. In contrast, starch-containing composites (e.g., S-30/5, S-40/5, and S-50/5) displayed more gradual and continuous release profiles, confirming that starch granules function as dissolution-triggered channels that facilitate controlled migration of antibacterial agents.

Multilayer structural designs have also been explored to further regulate release behavior. Ordoñez et al. [108] further demonstrated the effectiveness of multi-layered PLA/starch/PLA (PSP) films incorporating naturally derived antimicrobial agents such as ferulic acid and cinnamic acid. These films were engineered to leverage the complementary barrier properties of PLA and starch. Two surface incorporation strategies, spraying and electrospinning, were compared. While both methods imparted antimicrobial functionality, electrospinning yielded more effective encapsulation and release, as indicated by wider inhibition zones against Escherichia coli and Listeria innocua. The study revealed that film microstructure and additive incorporation method significantly influence the release rate and functional performance of active packaging systems.

**Figure 5:**
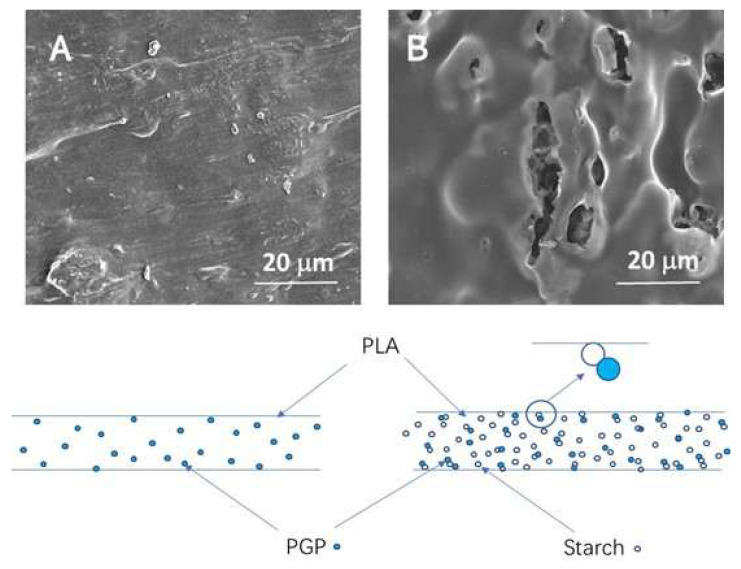
SEM surface images of (**A**) PLA/PGP and (**B**) PLA/starch/PGP coating and schematic representation of the microstructures [76].

**Figure 6:**
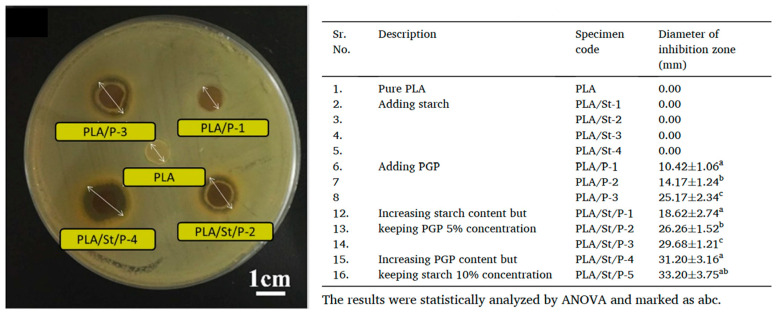
Typical inhibition zone of samples against *S. aureus* after 24 h of incubation at 37 °C for different samples [76].

Recently, Li et al. [76] used pomegranate peel (PGP) as both an antimicrobial and reinforcing agent to develop PLA/starch/PGP composite extrusion sheets for food packaging. It was found that the remarkable potential of PGP-infused sheets to inhibit the growth of Gram-positive bacteria, such as *S. aureus*. It is interesting to find that the starch particles not only act as a cheap filler but also act as a control release agent by providing channels for releasing functional substances from PGP contained in the PLA film. Even at lower PGP concentrations (5% *w*/*w*), the samples containing both starch and PGP exhibit significantly improved antimicrobial properties. As shown in Figure 5, PLA/PGP blends without starch displayed relatively smooth surfaces with embedded PGP particles fully encapsulated by the PLA matrix, limiting their release. In contrast, the addition of starch introduced structural discontinuities and visible microvoids. These channels increased the accessible surface area, improved permeability, and enhanced the release efficiency of PGP’s bioactive compounds. The release-enhancing role of starch was further confirmed by inhibition zone tests (Figure 6), where samples containing both PGP and starch (e.g., PLA/St/P-2 and P-4) exhibited significantly larger zones than their starch-free counterparts.

**Figure 7:**
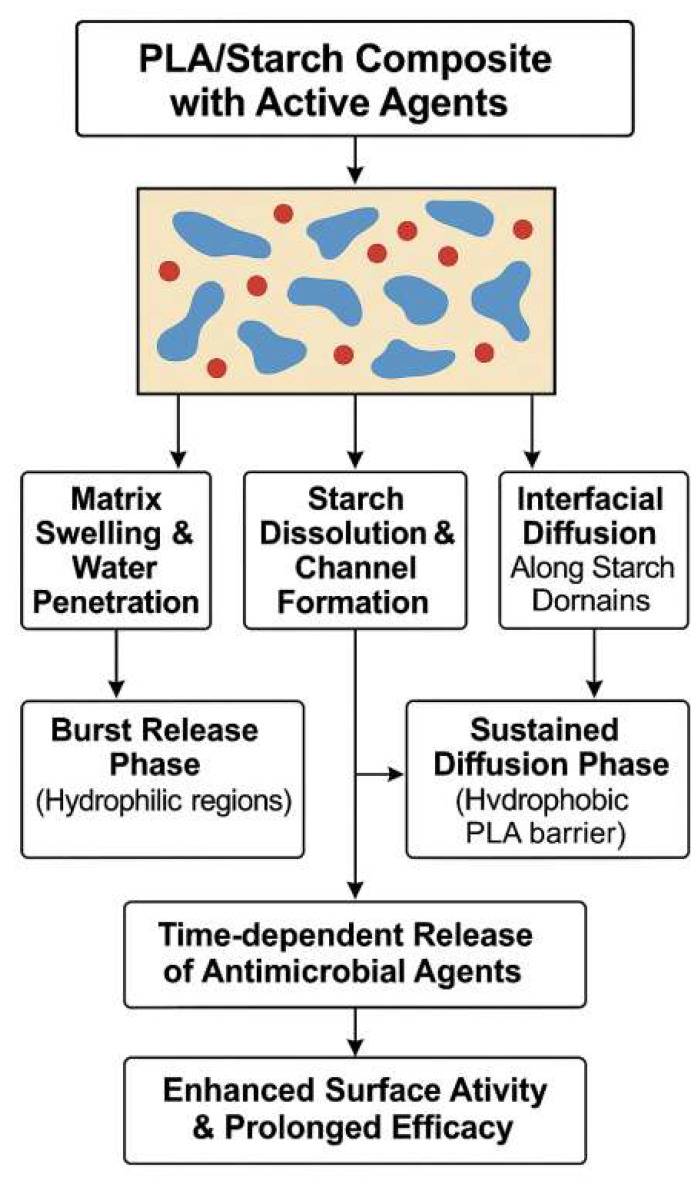
Schematic representation of controlled release mechanisms of antimicrobial agents in PLA/starch-based composite systems.

A schematic model of this mechanism is presented in Figure 7. The controlled release of antimicrobial agents from PLA/starch composites is governed by a combination of matrix and agent interactions and environmental triggers. Upon moisture exposure, the composite undergoes water penetration and swelling, particularly in starch-rich domains. Hydrophilic starch granules absorb water and partially dissolve, forming microchannels that facilitate agent diffusion. This initiates a two-phase release pattern: an initial burst release driven by water-soluble or surface-localized compounds, followed by a sustained release phase governed by the diffusion of agents through the more hydrophobic PLA matrix. Interfacial boundaries and phase-separated regions between PLA and starch further modulate the rate, directionality, and uniformity of agent transport. Together, these effects contribute to a prolonged and spatially targeted antimicrobial response.

## 4. Slow-Release Fertilizers Using PLA/Starch Composites

### 4.1. Development of Slow-Release Fertilizers

Conventional fertilizers suffer from significant inefficiencies, with up to 70% of nutrients being lost through processes such as decomposition, leaching, and ammonium volatilization, which occur in water, soil, and air. This inefficiency not only wastes valuable nutrients but also significantly contributes to environmental pollution, thereby affecting ecosystems and water quality. Moreover, the frequent and multi-periodic application of these fertilizers often leads to reduced fertilization efficacy over time, increasing the risk of soil and water toxicity and further stressing agricultural systems. These challenges have sparked a growing interest in the development and use of controlled-release and slow-release fertilizers (SRFs), particularly over the last two decades.

Slow-release fertilizers (SRF) technologies are designed to improve nutrient efficiency and minimize environmental impact by controlling the release of nutrients through chemical or physical mechanisms such as encapsulation, coating, and chemical modification. Traditional SRFs have primarily relied on petrochemical-derived polymers, sulfur-based compounds, and other synthetic materials to create nutrient-delivering coatings. However, increasing environmental concerns related to the persistence of non-biodegradable materials have driven a shift toward biodegradable alternatives. Recent efforts have focused on the development of biodegradable polymers, particularly those derived from renewable natural sources, such as starch, lignin, cellulose, chitosan, and alginate, as well as emerging nanocomposites. These materials offer a dual benefit: they maintain the controlled-release properties of traditional SRFs while being environmentally benign. By naturally more completely decomposing in soil, these biodegradable coatings reduce the risk of pollution and align with global sustainability goals. However, these SRFs coated with pure natural polymers either have higher costs or lower performance (mainly unstable) and are still in development.

Numerous studies have explored a wide range of coating materials for SRFs, including sulfur [118,119], polyolefin [120,121,122,123], polyurethane [124], and various natural polymers like chitosan [125,125], lignin [126,126], soybean oil [21] and starches [127,128,129,130,131,132,133] etc. Nevertheless, non-degradable synthetic coatings pose a long-term environmental threat due to their persistence in soil, prompting regulatory bodies to push for sustainable alternatives [134,135,136]. In light of tightening environmental regulations, biodegradable materials are increasingly considered not only desirable but potentially mandatory for future SRF applications [137].

Comprehensive reviews have categorized SRF technologies according to developmental stages, coating types, and release mechanisms [138,139,140,141,142,143,144,145,146,147,148]. In general, nutrient availability in SRFs can be controlled through two primary strategies: (1) delaying nutrient release within the soil matrix or (2) reducing solubility and diffusion in aqueous environments [127,149,150]. The actual release profile of an SRF is influenced by a variety of factors, including soil composition, crop type, temperature, and moisture levels. Various evaluation methods, such as soil column leaching tests, water extraction techniques, and incubation studies, have been developed to assess the performance of SRFs under different environmental conditions. As a result, reported release durations for the same formulation may range from hours to several months depending on the testing methodology used.

### 4.2. Biodegradable Coating Used for Slow-Release Fertilizers

The choice of coating materials is crucial in the development of effective slow-release fertilizers (SRFs), as different polymers exhibit distinct water vapor permeability (WVP) properties that directly influence nutrient release behavior. Devassine et al. [151] classified coating polymers into two primary categories: synthetic polymers and natural polysaccharides. Due to the abundance of hydrophilic hydroxyl groups in polysaccharides, natural polymers generally possess higher WVP coefficients compared to synthetic counterparts. Among the tested biopolymers, PLA and other biodegradable materials have demonstrated favorable barrier properties, making them promising candidates for SRF applications. From a materials design perspective, synthetic biodegradable polyesters, including PLA, polybutylene succinate (PBS), polybutylene adipate terephthalate (PBAT), and polycaprolactone (PCL), offer a balanced combination of mechanical strength, film-forming capability, and semi-permeability, rendering them suitable for controlled-release coatings. However, their practical applicability in agricultural systems is often limited by excessively slow biodegradation rates. These polymers typically require specific industrial composting conditions (e.g., high temperature and humidity) to fully decompose [14,16,152]. In contrast, agricultural standards, such as those defined by ISO, ASTM, and GB, require that coated fertilizers release at least 75% of their active nutrients within 27 days under natural soil conditions [153]. Unfortunately, full degradation of many synthetic biodegradable polyesters can take several months to years [154], rendering them unsuitable for practical use in SRFs aimed at natural degradation [155].

Among various encapsulation techniques, coating remains the most widely adopted and practical method for producing SRFs. Numerous studies have investigated the use of diverse coating materials [140]. More recently, Yuan et al. [146] reviewed the various oil coating used for developing SRFs. Materials used for coatings are generally classified into three categories: inorganic mineral compounds (gypsum, bentonite, sulfur [118], etc.), petroleum-based organic polymeric materials (polyolefins [120,122], polyethylene [123], polyurethane [156], polystyrene [121], super-absorbent polymer/hydrogel [157], polyacrylamide [158], polyacetal [159], polydopamine [160], etc.), and natural biodegradable polymeric materials (lignin [126], carboxymethyl cellulose [156], starch [159], and chitosan [125], etc.). Although sulfur-based coatings represent one of the earliest commercial SRF technologies [161,162,163,164], they have gradually fallen out of favor due to high production costs, brittleness, poor coating uniformity, and inconsistent release behavior [144,165].

**Figure 8:**
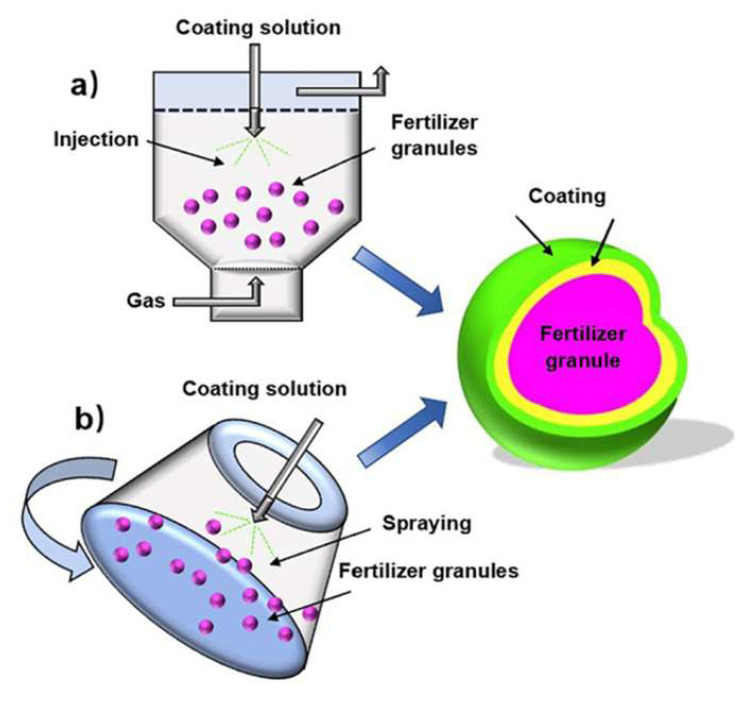
Schematic representation of coating by (**a**) a rotary drum coater; (**b**) a fluidized bed coater [136].

For highly water-soluble fertilizers such as urea, coating layers are designed to function as semipermeable barriers that delay water infiltration and regulate nutrient diffusion through osmotic- and capillary-driven mechanisms [166]. The release behavior is therefore governed not only by coating composition but also by environmental factors such as soil moisture, microbial activity, coating thickness, and temperature, which act synergistically to influence nutrient transport [161]. To accommodate large-scale production, commercial SRF manufacturing commonly employs pan coaters, rotary drum coaters, and fluidized bed coating systems [151], as schematically illustrated in Figure 8.

Against this background, the integration of hydrophilic polysaccharides such as starch into PLA-based coatings has emerged as a rational design strategy. In such composite systems, PLA provides the structural framework and barrier function, while starch introduces controlled permeability and water responsiveness. Importantly, the intrinsic immiscibility between PLA and starch leads to phase-separated microstructures that can be deliberately exploited to regulate water transport and nutrient diffusion. This synergistic design concept forms the basis for PLA/starch composite coatings discussed in the following section, where controlled release is achieved by balancing barrier integrity and water-triggered channel formation.

### 4.3. Application of PLA/Starch Coating for Developing SRFs

Recent studies have demonstrated that PLA/starch composite coatings offer a viable and sustainable strategy for developing slow-release fertilizers (SRFs) by integrating the complementary barrier and water-responsive properties of the two components. Li et al. [20] reported the development of a sustainable coating system based on PLA and rice starch, wherein starch granules served dual roles, as economical fillers and as hydrophilic agents facilitating nutrient release. By tuning the PLA-to-starch ratio, the rate of water channel formation within the coating matrix could be precisely modulated, thereby enabling controlled urea release. The study also demonstrated that the morphological and thermal properties of the coatings were strongly influenced by starch content.

**Figure 9:**
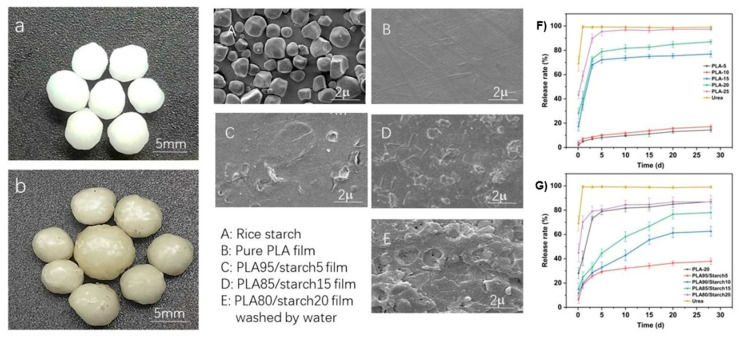
Photos of native (**a**) and coated (**b**) urea particles, SEM images of coating surfaces (**A**–**E**) and nitrogen release rate from (**F**) PLA coating and (**G**) PLA/starch coating [20].

As illustrated in Figure 9, photographic and scanning electron microscopy (SEM) analyses revealed distinct morphological differences between uncoated and coated urea particles. Native urea granules exhibited smooth, spherical surfaces, whereas PLA/starch-coated particles showed a uniform, continuous shell with a slightly increased particle size and yellowish appearance, indicating effective encapsulation. SEM images of the coating surface further revealed pronounced roughness, characterized by elevated ridges and “hill-like” features corresponding to embedded starch granules within the PLA matrix. These microstructural heterogeneities closely resemble those observed in PLA/starch composite films and are widely regarded as precursors to water-triggered microchannels that facilitate water ingress and nutrient diffusion during release. Nitrogen release profiles (Figure 9) clearly demonstrate the critical influence of coating composition on release behavior. Pure PLA coatings substantially suppressed nitrogen release compared with uncoated urea, confirming the excellent barrier performance of PLA. However, at low PLA concentrations (PLA-5, PLA-10, and PLA-15), incomplete coating coverage resulted in rapid early-stage nitrogen release, producing release profiles similar to those of uncoated urea. In contrast, when the PLA concentration reached 20% or higher, a continuous and defect-free coating was formed, leading to a dramatic reduction in cumulative nitrogen release, which stabilized at approximately 14% over 27 days. This excessively low release rate explains why many biodegradable polyesters, despite their favorable mechanical properties, are rarely used alone as SRF coatings. Consequently, a PLA concentration of 20% was selected as the baseline formulation to ensure sufficient coating integrity.

Compared with pure biodegradable polyester coatings, which often exhibit overly restrictive permeability, PLA/starch composites provide a wider and more tunable release window by decoupling structural integrity from water transport pathways [167]. This behavior reflects the opposing roles of the two components: the relatively high crystallinity of PLA forms a dense barrier that effectively suppresses water penetration and nutrient diffusion, whereas starch domains introduce water-responsive discontinuities that transform into diffusion channels, thereby enabling tunable release profiles in PLA/starch composite coatings. As starch content increased, nitrogen release rates rose correspondingly, primarily due to the gradual hydration and partial dissolution of starch granules, which generated pores and diffusion pathways within the coating. Importantly, the relationship between starch content and nutrient release was highly nonlinear. At low starch loading (PLA95/Starch5), the release rate stabilized at approximately 30% after five days, indicating moderate permeability. In contrast, excessive starch content (PLA80/Starch20) led to rapid burst release, comparable to that of uncoated urea, as excessive channel formation compromised the integrity of the barrier at an early stage. An optimal release profile was achieved at intermediate starch contents (PLA90/Starch10 and PLA85/Starch15), where nitrogen release proceeded in a smooth and sustained manner without premature nutrient loss. In this compositional window, the barrier function of PLA and the water-responsive behavior of starch were effectively balanced, yielding release kinetics that closely meet the agronomic requirements for SRFs. These results indicate that a starch content of approximately 10–15% represents a critical design range for achieving predictable and controllable nutrient release. It should be noted, however, that nutrient release behavior in real soil environments is strongly influenced by moisture fluctuations, microbial activity, and soil texture, which may alter the dissolution kinetics of starch-rich domains and, consequently, the long-term release profile [155].

**Figure 10:**
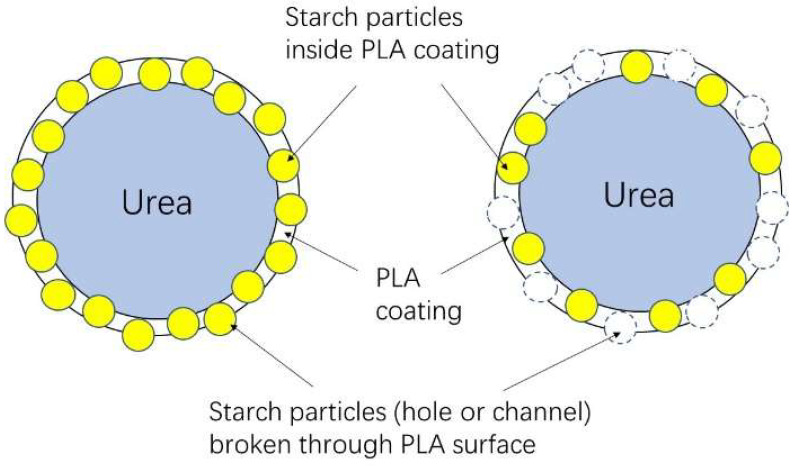
Schematic representation of developed SRF through coating urea by PLA/starch composites, in which some starch particles could act as channels for urea releasing after dissolving in water [20].

The underlying release mechanism is schematically illustrated in Figure 10. Upon exposure to moisture, hydrophilic starch domains embedded within the PLA coating preferentially absorb water and partially dissolve, forming interconnected microchannels that facilitate the diffusion of urea from the fertilizer core. This water-triggered channel formation mechanism is analogous to that observed in PLA/starch-based antimicrobial packaging systems, underscoring a unified structure–function principle across food and agricultural applications. By exploiting the intrinsic phase separation between PLA and starch, composite coatings can thus transform a traditional compatibility limitation into a functional advantage, enabling the development of efficient, environmentally friendly slow-release fertilizers.

### 4.4. Critical Challenges and Research Outlook

Despite promising progress, several challenges must be addressed before PLA/starch-based SRFs can be widely adopted. First, the long-term mechanical integrity of composite coatings under cyclic wet–dry soil conditions remains insufficiently understood. Second, nutrient release behavior is highly sensitive to soil moisture, temperature, and microbial activity, necessitating standardized evaluation protocols to enable meaningful comparison across studies. Third, large-scale processing, cost competitiveness, and coating uniformity under industrial conditions require further optimization. Future research should therefore focus on (i) establishing quantitative relationships between starch microstructure, coating morphology, and release kinetics; (ii) integrating multi-nutrient or multi-functional coatings; and (iii) validating performance under realistic field conditions. Addressing these issues will be essential for translating PLA/starch composite coatings from laboratory concepts into practical, sustainable solutions for modern agriculture.

## 5. Concluding Remarks and Future Perspectives

PLA/starch composites represent a promising class of biodegradable materials owing to their renewable origins and complementary advantages, including reduced material cost, enhanced biodegradability, and tunable moisture sensitivity. Nevertheless, intrinsic immiscibility and weak interfacial adhesion between PLA and starch often lead to compromised mechanical and barrier properties, limiting their broader application. To overcome these challenges, strategies such as starch surface modification and PLA functionalization have been widely employed to improve interfacial compatibility, thereby enhancing the structural integrity, water resistance, and long-term stability of PLA/starch composites.

Beyond conventional applications, increasing attention has been directed toward the use of PLA/starch composites as controlled-release materials. Their inherent phase-separated morphology and the distinct water solubility and degradation behaviors of PLA and starch enable starch-rich domains to function as water-responsive channels within the PLA matrix. By tailoring starch content and distribution, the release profiles of functional agents can be effectively regulated. This concept has been successfully demonstrated in antimicrobial food packaging systems (e.g., PLA/starch composites incorporating plant-derived antimicrobial agents) and in slow-release fertilizer coatings, where PLA/starch layers modulate the diffusion of nutrients such as urea. Overall, the reported studies highlight the versatility of PLA/starch composites as sustainable and multifunctional materials.

Despite these advances, several challenges remain that warrant further investigation. Future research should focus on establishing quantitative structure–function relationships linking starch microstructure, interfacial architecture, and release behavior under realistic service conditions. Greater attention is also needed to standardize testing protocols for biodegradability, barrier properties, and release performance to enable meaningful comparison across studies. Moreover, long-term performance and degradation behavior in real food packaging and soil environments remain insufficiently explored. Addressing these challenges will be critical for translating laboratory-scale developments into practical applications. Overall, continued progress in interface design, microstructural control, and application-oriented evaluation is expected to further expand the role of PLA/starch composites as sustainable, multifunctional materials aligned with circular economy principles and resilient food–agriculture systems.

## Figures and Tables

**Table 1:** Summary of antimicrobial agents incorporated in PLA-based composite systems.

Antimicrobial Agent	Origin/Type	Composition	Target Microorganisms	Antimicrobial Performance	Application Scenario	Reference
Nisin	Natural bacteriocin	PLA film (no starch)	*Lactobacillus plantarum*	Complete inhibition at 1.0 mg/cm^2^	Ready-to-eat foods	[74]
Pomegranate peel extract	Plant polyphenols	PLA/Starch composite	*S. aureus*, *E. coli*	Inhibition rate > 99.9%	Active food packaging	[76]
Nisin (Nisaplin^®^)	Natural bacteriocin	PLA bilayer film	*Listeria monocytogenes*	>5.0 log CFU/mL reduction	Meat and poultry packaging	[77]
Nisin-Pectin Microparticles	Bacteriocin in carrier	PLA composite	*Listeria monocytogenes*	~4.0 log CFU/mL reduction	Antimicrobial packaging	[78]
Triclosan	Synthetic broad-spectrum	PLA/Wood Flour composite	*Escherichia coli*	Inhibition zone: 1.8–7.0 mm	General food packaging	[103]
Triclosan/β-CD Complex	Inclusion complex	Electrospun PLA nanofibers	*E. coli*, *S. aureus*	Distinct inhibition zones	Active packaging webs	[105]
Thymol + AgNPs	Phenolic + Nanomaterial	PLA nanocomposite	Bacteria (general)	Antibacterial confirmed	Multi-functional packaging	[106]
Chitosan	Natural biopolymer	PLA/Starch/Chitosan blend	*E. coli*, *S. aureus*	Moderate inhibition	Biodegradable films	[107]
Ferulic/Cinnamic Acid	Phenolic acids	PLA/Starch	*S. aureus*, *E. coli*, *Penicillium expansum*	Inhibition zones: 1.2–2.5 cm	Multilayer active packaging	[108]

**Table 2:** Summary of food packaging applications PLA/starch-based composite films.

Composition	Structure Type	Application Scenario	Application Effect	Reference
Pea starch + PLA	Bilayer film	Cherry tomato packaging	Delayed softening, discoloration, and decay; extended shelf life by 3–5 days	[109]
PLA + Corn starch	Biocomposite film	Fresh produce packaging	Enhanced gas barrier properties; extended freshness period by 30%	[113]
Avocado seed starch + PLA	Biocomposite film bacteriocin	sunflower seed	Reduced photo-oxidative degradation by >50%	[114]
Modified porous starch + PLA/PBAT	Biocomposite film	High-fat food packaging	Reduced microbial count by 62%; inhibited lipid oxidation by 48%	[110]
Pea starch + PLA	Sandwich-architectured film	Strawberry preservation packaging composite	Extended freshness retention by 2–3 days compared to bilayer films	[115]
Rice straw fractions + PLA-starch bilayer	Reinforced bilayer film	Refrigerated pork packaging	reduced rancidity rate by 45%	[111]
PLA + starch bilayer	bilayer film	PLA Fresh vegetable packaging	Inhibited E. coli and Aspergillus niger; reduced microbial colony count by >60%	[112]
Starch-based PLA/PBAT + Natural antimicrobials	Biocomposite film	Beef refrigerated packaging	Extended shelf life by 6–8 days under refrigeration	[116]

## Data Availability

No new data were created or analyzed in this study. Data sharing is not applicable to this article.
